# Dual Grain Refinement Effect for Pure Aluminum with the Addition of Micrometer-Sized TiB_2_ Particles

**DOI:** 10.3390/ma17174337

**Published:** 2024-09-02

**Authors:** Ke Wang, Chunfang Zhao, Yihuan Song, Mingjie Wang, Fei Wang

**Affiliations:** 1 School of Mechanical and Electrical Engineering, Lishui Vocational and Technical College, Lishui 323000, China; zhaochunfang0710@126.com; 2 Shenzhen Institute of Advanced Technology, Chinese Academy of Sciences, Shenzhen 518055, China; syh9999999@126.com; 3 Captial Spaceflight Machinery Company Co., Ltd., Beijing 100071, China; mingjie_12138@163.com; 4 School of Material Science and Chemical Engineering, Harbin University of Science and Technology, Harbin 150080, China; wfambitious@126.com

**Keywords:** grain refinement, TiB_2_ particles, pure aluminum, heterogeneous nucleation

## Abstract

The inefficiency of grain refinement processes has traditionally been attributed to the limited utilization of heterogeneous nucleation particles within master alloy systems, resulting in the formation of abundant inactive particles. This study aims to investigate the alternative influences of particles by incorporating external micrometer-sized TiB_2_ particles into the grain refinement process. Through a series of experiments, the refinement efficiency, grain refinement mechanism, and resultant microstructure of TiB_2_ particle-induced grain refinement specimens are comprehensively examined using various microscopy and analytical techniques, including polarization microscopy (OM), scanning electron microscopy (SEM), differential scanning calorimetry (DSC), and transmission electron microscopy (TEM). Our findings demonstrate a direct correlation between increased levels of TiB_2_ particles and enhanced grain refinement efficiency. Moreover, the microstructure analysis reveals the distribution of TiB_2_ particles along grain boundaries, forming a coating due to self-assembly phenomena, while regions with a lower particle content may exhibit irregular grain structures. DSC analysis further confirms reduced undercooling, indicating the occurrence of heterogeneous nucleation events. However, TEM observations suggest that heterogeneous nucleation is not significantly influenced by the growth restriction factor attributed to TiAl_3_ 2DC compounds. The grain refinement mechanism involving TiB_2_ particles is elucidated to entail both heterogeneous nucleation and physical growth restriction effects. Specifically, a reduction in average grain size is attributed not only to heterogeneous nucleation but also to the physical growth restriction effect facilitated by the TiB_2_ particle coating. This study offers insights into leveraging particles that do not participate in heterogeneous nucleation within master alloy-based grain refinement systems.

## 1. Introduction

Aluminum and its alloys are highly valued in industrial manufacturing because of their advantageous combination of low density and high specific strength [1]. In both the deformation and casting processes of aluminum alloys, grain refinement holds paramount importance, as it leads to a significant reduction in shrinkage and enhances mechanical as well as processing properties. The topic of grain refinement in aluminum alloys has been extensively studied over many decades, with a primary focus on introducing diverse materials such as the Al-5Ti-1B master alloy and nano-TiC particles to induce the heterogeneous nucleation of α-Al [2,3].

In practical applications, the Al-5Ti-1B master alloy dominates approximately 75% of the market due to its ability to offer a robust growth restriction factor and efficient TiB_2_ heterogeneous nucleation particles [4,5]. Through the synergistic effects of these properties, the grain size of α-Al can typically be refined to approximately 200 μm. Nevertheless, extensive experience over decades has revealed that enhancing grain refinement beyond this point poses a significant challenge. The primary obstacles to this include the scarcity of heterogeneous nucleation particles and the complexity of improving growth restriction factors.

The free growth model reveals the critical size, d, of heterogeneous nucleation particles as a function of undercooling [6,7]:

(1) $$d=\frac{4\sigma }{\Delta T_{fg}\Delta S_{V}}$$

where σ represents the solid–liquid interfacial energy, $\Delta S_{V}$ is the entropy of fusion per unit volume, and $\Delta T_{fg}$ represents undercooling. This implies that particles smaller than the critical size are available to contribute to grain refinement. Furthermore, research has shown that the heterogeneous nucleation of α-Al exhibits significant dependence on crystal plane orientation, with nucleation primarily occurring on the (0001) surface of TiB_2_ within the Al/Al-Ti-B grain refinement system [8,9]. TiB_2_ particles unable to present a suitable (0001) surface during nucleation events remain inactive during solidification.

The stringent criteria governing heterogeneous nucleation events lead to a substantial proportion of TiB_2_ particles remaining inactive. Effectively harnessing these particles could offer a solution for the development of more efficient master alloys. Recent studies have demonstrated that the average grain size of pure aluminum can be noticeably refined by incorporating Ti(C 0.7, N 0.3) particles using an Al-4.2 wt.% Ti(C 0.7, N 0.3) master alloy [10]. Furthermore, the significant grain refinement of pure aluminum has been achieved through the addition of pure TiCN nanoparticles [11]. These findings suggest a promising pathway for grain refinement through the use of pure particles and the effective utilization of idle particles via master alloy addition methods.

To the best of our knowledge, previous research on grain refinement primarily focused on nano-sized particles, with limited attention given to micron-sized particles. However, TiB_2_ particles, which are approximately 10 μm in size, are commonly utilized as heterogeneous nucleation agents in the Al-5Ti-1B master alloy. This study aims to explore the feasibility of grain refinement through the addition of micron-sized particles. Our objective is to pave the way for valuable research opportunities concerning the utilization of particles that do not participate in heterogeneous nucleation at the micrometer scale.

## 2. Materials and Method

Grain refinement was conducted on commercially pure aluminum (CPAl) (purity > 99.9 wt.%) obtained from a commercial supplier. Commercially pure TiB_2_ particles (purity > 99.9 wt.%) with an average size of 10 μm were employed as the grain refiner. All grain refinement experiments were conducted under consistent conditions throughout this study.

The process began by melting pure aluminum at a temperature of 760 °C and subjecting it to refining using Cl_2_C_6_ for a duration of 10 min. Subsequently, TiB_2_ particles, enclosed within aluminum foil, were introduced into the aluminum melt and thoroughly stirred using a graphite rod to ensure the proper dispersion of the added particles. The molten mixture was then held in the furnace for 20 min. Following the removal of slag, the aluminum melt containing TiB_2_ particles was poured into steel molds with dimensions of 38 mm in diameter and 30 mm in height. Prior to pouring, the aluminum melt was stirred with a graphite rod for 5 s. Additionally, a comparative grain refinement experiment was conducted under identical conditions by adding 0.2 wt.% of the Al-5Ti-1B master alloy.

The bottom surfaces of the refined specimens were ground, polished, and etched using Poulton’s reagent to reveal macroscopic grains. Standard metallographic procedures were followed, and the microstructure was observed using an Olympus DSX1000 (Tokyo, Japan) digital microscope (OM). Samples etched with Barker’s reagent were examined using a Zeiss Axio VertA1 (Oberkochen, Germany) polarized light optical microscope (OM). The average grain size of pure aluminum was determined using the linear intercept method in accordance with ASTM E112-13 (2021) [12]. Microstructural analysis of the refined specimens was performed using a Hitachi SU 8010 (Tokyo, Japan) scanning electron microscope (SEM) equipped with an Oxford Energy Dispersive Spectrometer (EDS) (Oxford, UK). X-ray diffraction (XRD) analysis of the pure aluminum ingots refined with TiB_2_ particles was conducted using a Rigaku Smart Lab 3kW X-ray diffractometer (Tokyo, Japan) with Cu-Kα radiation (40 kV, 40 mA) at a scanning speed of 4 degrees per minute. Furthermore, the thermal analysis of pure aluminum with TiB_2_ particles was carried out using a Netzsch STA 449F3 (Selb, Germany) differential scanning calorimeter (DSC) at a heating and cooling rate of 10 K/min under argon gas protection at a flux rate of 20 mL/min. The temperature range was set to 25–750 °C. The samples used in TEM were prepared by the Gatan 691 (Warrendale, PA, USA) ion-thinning instrument. A JEM-F200 (Tokyo, Japan) transmission electron microscopy/high-resolution transmission electron microscopy (TEM/HRTEM) was employed to study the phase boundary of α-Al/TiB_2_ at a nanometer scale.

## 3. Results

**Figure 1:**
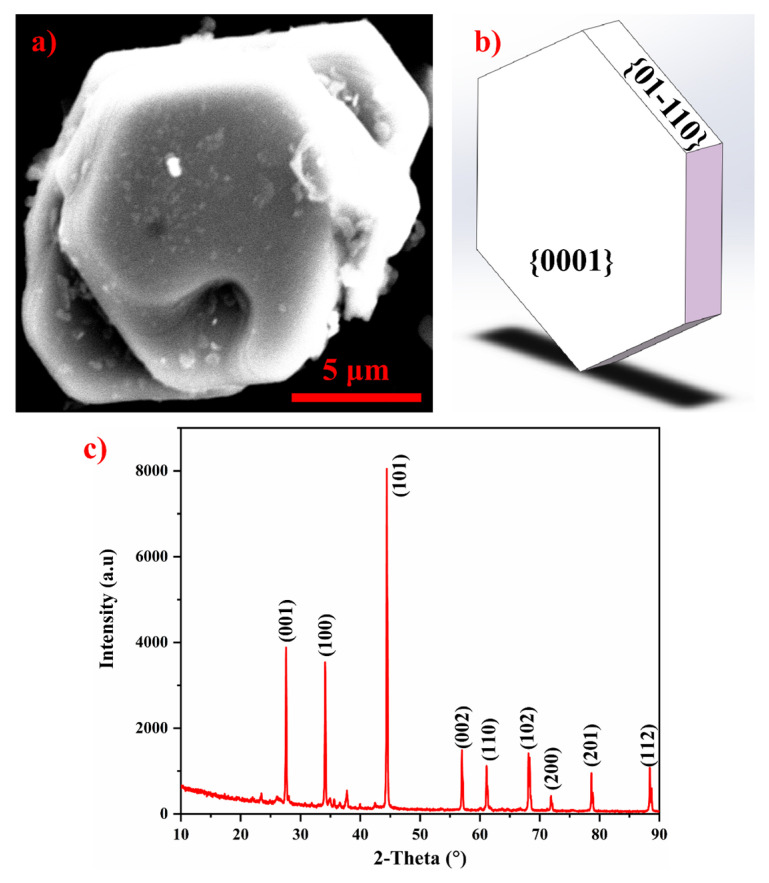
Morphology of TiB_2_ particles. (**a**) TiB_2_ particles used in this experiment, (**b**) schematic illustration of in situ TiB_2_ particles, and (**c**) XRD pattern of TiB_2_ particles.

Figure 1 illustrates the scanning electron microscope (SEM) morphology of the TiB_2_ particles employed in this study. As depicted in Figure 1a,b, the TiB_2_ particles exhibit a plate-like morphology with an average size of approximately 10 μm (consideration: approximate the size of in situ particle agglomeration), differing in morphology from in situ TiB_2_ with the hexagonal platelets found in the Al-5Ti-1B master alloy [13,14]. The X-ray diffraction (XRD) pattern of TiB_2_ particles, displayed in Figure 1c, confirms that the raw material meets AR (as-received)-grade specifications, which is crucial for ensuring the integrity of the experimental investigation.

**Figure 2:**
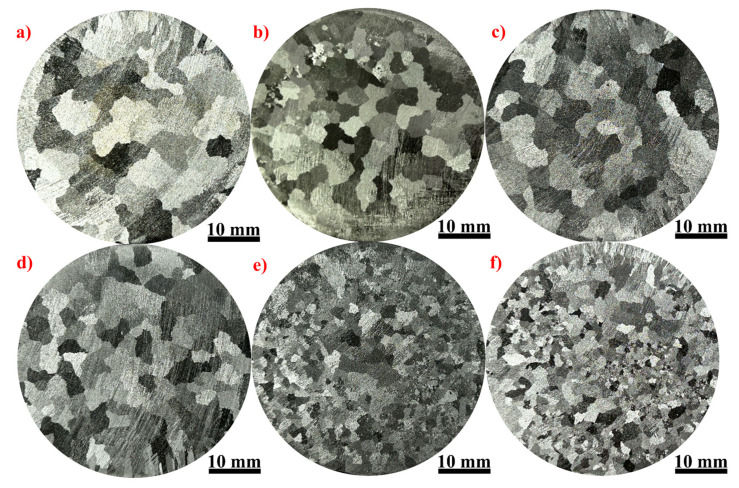
Macrostructure grains of pure aluminum refined by various TiB_2_ addition levels. (**a**) 0 wt.%; (**b**) 0.05 wt.%; (**c**) 0.1 wt.%; (**d**) 0.2 wt.%; (**e**) 1.0 wt.%; and (**f**) 5.0 wt.%.

**Figure 3:**
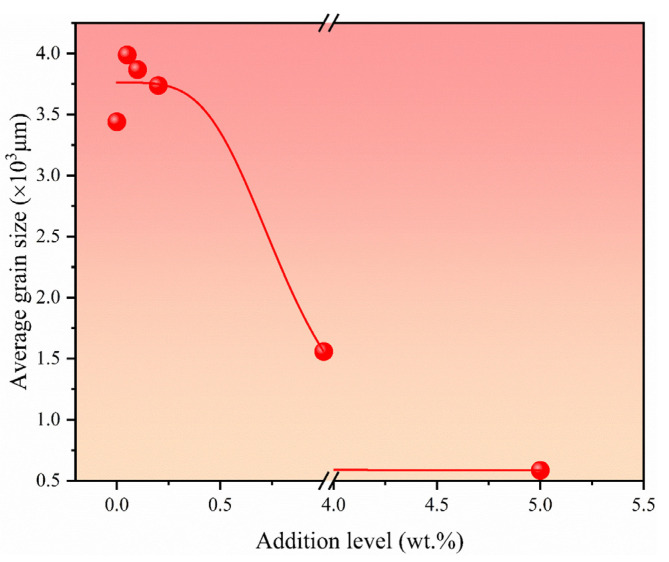
Average grain size of pure aluminum refined by various TiB_2_ addition levels.

Figure 2 presents the typical macrostructure grains of as-cast pure aluminum, both without and with various additions of TiB_2_ particles. In the microstructure of pure aluminum depicted in Figure 2a, coarse equiaxed grains are predominant. However, as depicted in Figure 2b–d, the addition of TiB_2_ particles leads to a slight reduction in the average grain size of pure aluminum. Specifically, the grain size of pure aluminum exhibits minimal change with TiB_2_ additions below 1.0 wt.%. Nonetheless, noticeable reductions in grain size are observed at addition levels of 1.0 wt.% and 5.0 wt.%, albeit with a notable degree of variation, as depicted in Figure 3, illustrating the trend of grain size with varying TiB_2_ addition levels. Figure 3 demonstrates that the average grain size decreases by 66.33% and 75.93% at respective addition levels of 1.0 wt.% and 5.0 wt.%. These grain refinement experiments underscore the extreme sensitivity of the grain size of pure aluminum to the level of TiB_2_ addition. These compelling results affirm the effectiveness of reducing the average grain size of pure aluminum through the addition of external TiB_2_ particles.

**Figure 4:**
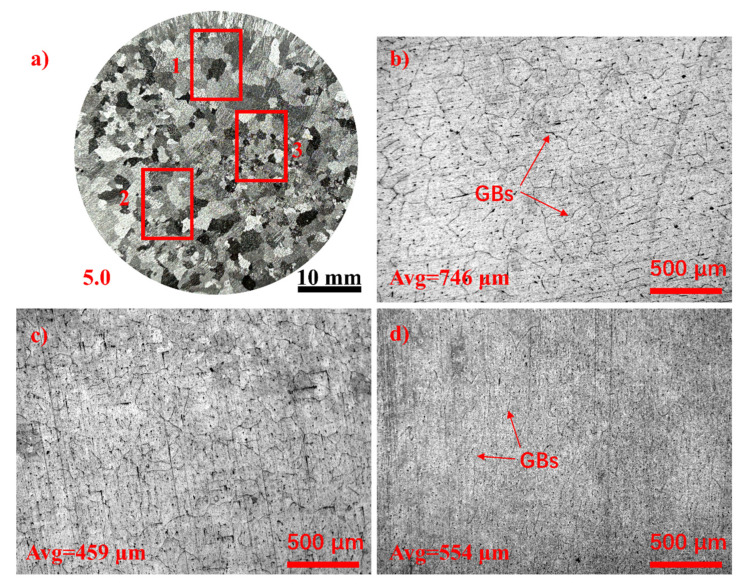
Microstructure grain differences in pure aluminum refined by 5.0 wt.% TiB_2_. (**a**) Macrostructure grains; (**b**) microstructure grains of area 1; (**c**) microstructure grains of area 2; and (**d**) microstructure grains of area 3.

Figure 4 depicts the variation in grain size within the 5.0 wt.% specimen. As shown in Figure 4a, areas 1 to 3 exhibit distinct differences in grain size locally, demonstrating that the addition of TiB_2_ particles can result in varying average grain sizes within a single sample. The corresponding microstructures of these areas are depicted in Figure 4c,d. Statistical analysis reveals that area 1 exhibits the largest average grain size of approximately 746 μm, while area 2 displays the smallest average grain size of approximately 459 μm. Additionally, the presence of numerous winding and narrow grain boundaries (GBs) in area 3, as observed in Figure 4d, stands out prominently compared to Figure 4b,c. This characteristic is distinct from the features typically associated with the grain refinement induced by in situ heterogeneous nucleation particles, such as certain master alloys containing TiB_2_ particles in Al-Ti-B and TiC particles in Al-Ti-C [15,16,17].

**Figure 5:**
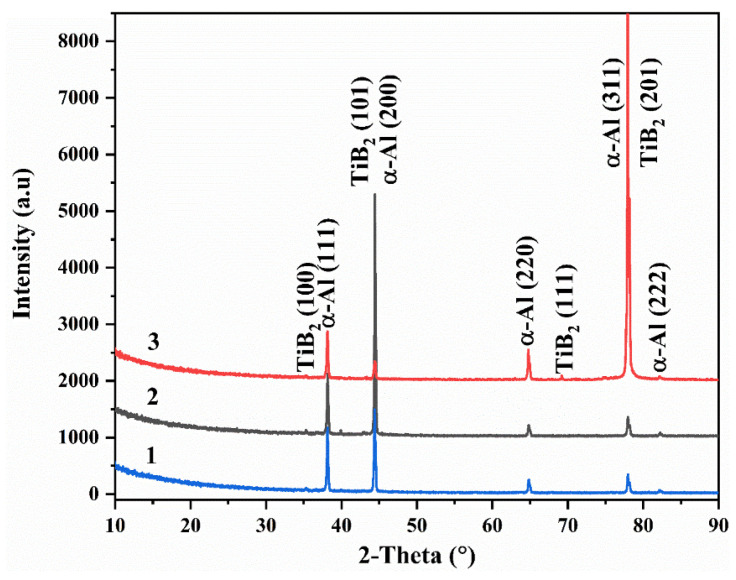
XRD patterns of areas 1~3.

Figure 5 presents the X-ray diffraction (XRD) patterns of areas 1 to 3. In area 1, as depicted in the pattern, the larger grain size of pure aluminum corresponds to a lower concentration of TiB_2_ particles, as evidenced by a faint (100) reflection. Conversely, the pattern of area 2 exhibits a prominent peak at around 44°, primarily attributed to the (101) reflection of TiB_2_ and the (200) reflection of α-Al. This increase in peak intensity reflects the higher concentration of TiB_2_ particles in area 2, correlating with a reduction in the average grain size. Notably, the number of TiB_2_ particles is inferred to be more substantial in area 3 due to the smaller average grain size, which is a deduction supported by the XRD pattern. However, an intriguing observation arises from the fact that the most intense reflection of the added TiB_2_ particles shifts from the original (101) to the (201) crystal face, contradicting the (101) preferred orientation indicated by the powder XRD pattern.

**Figure 6:**
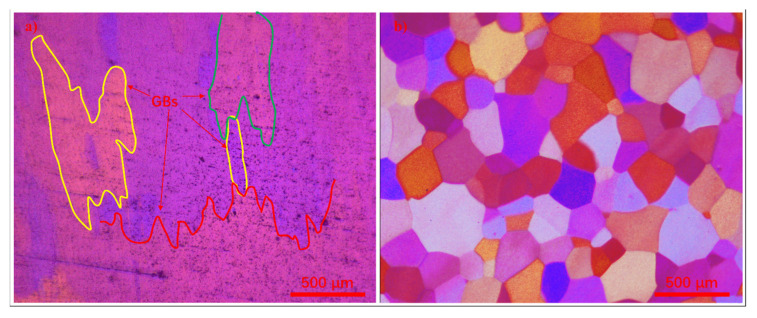
Microstructure grains of pure aluminum refined by (**a**) TiB_2_ particles and (**b**) the Al-5Ti-1B master alloy.

To further elucidate the grain boundary characteristics, Figure 6 compares the grain boundary morphology of refined pure aluminum achieved through TiB_2_ particles and the Al-5Ti-1B master alloy. As illustrated in Figure 6a, the grain boundaries exhibit pronounced irregular and serrated morphology, resulting in grains of varied shapes that interact with their surroundings. In contrast, the grain morphology influenced by Al-5Ti-1B displays smoother characteristics with clearly defined grain boundaries and no evidence of interlocking between grains. Figure 6 suggests that the mechanism of grain refinement utilizing particles and master alloys may originate from distinct mechanisms, even within a single sample, where the anisotropy differs from the isotropic refinement achieved by the master alloy.

**Figure 7:**
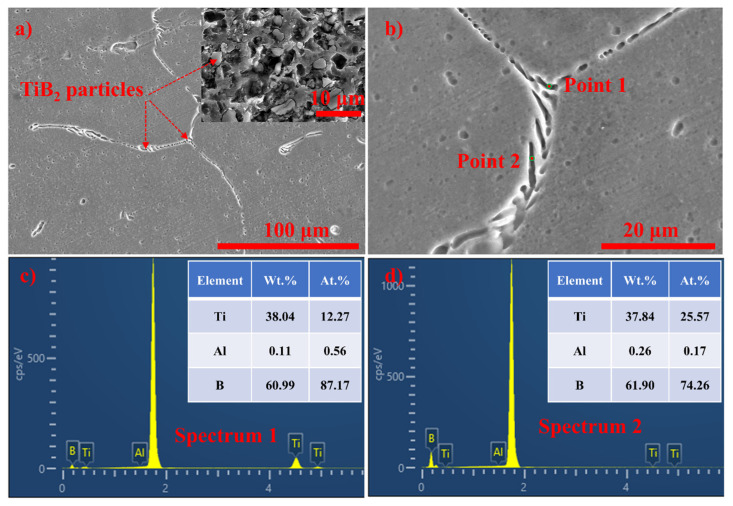
SEM micrographs of pure aluminum refined by 5.0 wt.% TiB_2_. (**a**) A low magnification SEM micrograph inset is deeply etched in the microstructure; (**b**) high-magnification SEM micrograph; (**c**) EDS spectrum 1 detected from point 1; and (**d**) EDS spectrum 2 detected from point 2.

Figure 7 depicts the distribution of TiB_2_ particles within refined pure aluminum. As shown in Figure 7a,b, the TiB_2_ particles are predominantly located at the grain boundaries, with some penetrating into the grain interiors. The concentration of TiB_2_ particles is relatively high. During the growth of α-Al grains, a significant number of TiB_2_ particles accumulate at the leading edge of the grain solidification interface. Some particles become embedded within the growing grains and are transported into the grain interiors, while others are pushed forward. As the grains continue to grow, these particles accumulate along the grain boundaries.

## 4. Discussion

Previous studies have reported that the mere addition of ~1 part per thousand (p.p.t) of the Al-5Ti-1B master alloy can effectively reduce the average grain size of pure aluminum to approximately 200 μm [6]. Despite our efforts to focus on TiB_2_ particles that closely resemble in situ conditions as the primary research focus, our results still exhibit variations compared to the Al-5Ti-1B master alloy in terms of grain refinement efficiency and the morphology of refined grains.

The grain size prediction model demonstrates the volume density of active nucleation sites, the growth restriction factor, and undercooling, which dominates the final grain size as shown in the following expression:

(2) $$d=\frac{1}{\sqrt[{3}]{N}}+\frac{b\cdot \Delta T}{Qv}$$

**Figure 8:**
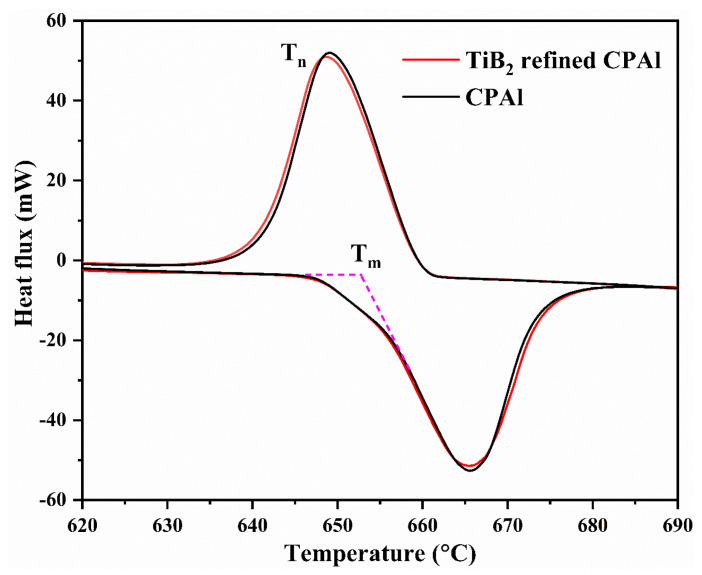
DSC analysis of pure aluminum without and with 5.0 wt.% TiB_2_ particles.

Here, N is the volume density of active nucleation sites, ΔT represents undercooling, Q is the growth restriction factor, v is the cooling rate, and b is the constant. In this experiment, the factors influencing the final grain size of pure aluminum primarily revolve around active nucleation sites, given the consistent experimental conditions. The anticipated dramatic decrease in the average grain size of pure aluminum with increasing levels of TiB_2_ particle addition did not materialize as expected. Previous research has demonstrated that the addition of master alloys for grain refinement effectively reduces nucleation undercooling compared to unrefined systems [18]. Figure 8 illustrates the undercooling (Δ*T*) of pure aluminum with and without the addition of TiB_2_ particles, as measured using differential scanning calorimetry (DSC) technology during solidification. The undercooling between pure aluminum and 0.5 wt.% TiB_2_ particles of refined pure aluminum exhibited a slight discrepancy when calculated using the method referenced in [19]. According to the DSC results, the undercooling of pure aluminum without and with 0.5 wt.% TiB_2_ particles are approximately 7.55 °C (melting temperature: 651.43 °C; nucleation temperature: 643.88 °C) and 7.52 °C (melting temperature: 650.99 °C; nucleation temperature: 643.47 °C), respectively, which is consistent with the reported suggestion that undercooling can be as low as 0.1 K [20]. These DSC results provide substantial evidence that TiB_2_ particles indeed participate in heterogeneous nucleation events. Specifically, the induction of heterogeneous nucleation events by adding TiB_2_ particles effectively validates the reduction in grain size and the formation of regular grain boundaries.

**Figure 9:**
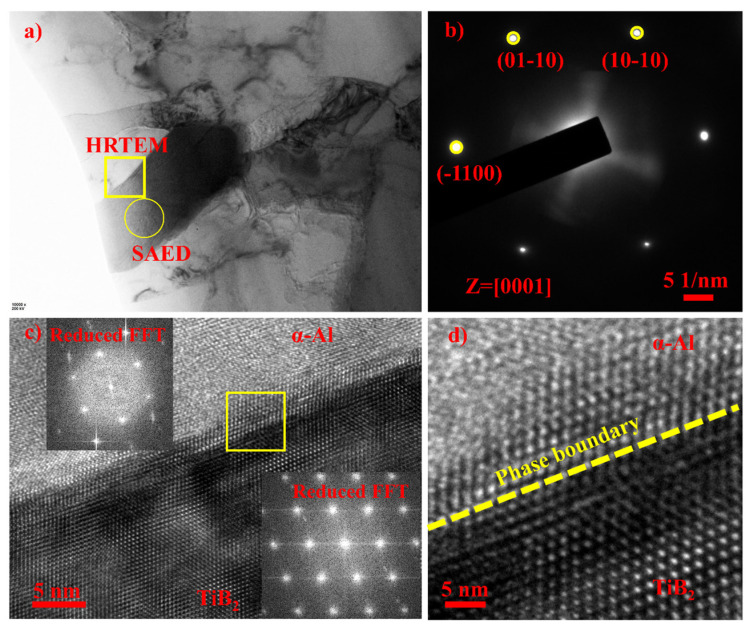
TEM analysis of α-Al/TiB_2_ phase boundary. (**a**) BF image of α-Al/TiB_2_ phase, (**b**) SAED pattern of TiB_2_ phase detected from ring in (**a**), (**c**) HRTEM image of α-Al/TiB_2_ phase boundary coming from square frame in (**a**); insets are reduced FFT images of α-Al and TiB2 phase, respectively. (**d**) Enlarged phase boundary image of α-Al/TiB_2_ coming from square frame in (**c**).

Solute elements play a critical role in heterogeneous nucleation events, with their growth restriction effect significantly enhancing nucleation on heterogeneous sites [5,7,21]. The prevalence of Ti-containing master alloys in the grain refinement industry can be attributed to the high-growth restriction factor of titanium [22]. Free titanium atoms facilitate the formation of a thin (112) TiAl_3_ two-dimensional compound (TiAl_3_ 2DC) on the surfaces of TiB_2_ particles, thereby promoting effective grain refinement by reducing lattice misfit between the sites and α-Al [23,24]. In this experiment, Ti atoms from TiB_2_ particles served as the sole titanium source and were released through dissolution after addition [25]. However, the amount of released Ti was significantly lower than the critical value of 0.15 wt.% required for the peritectic reaction of TiAl_3_ 2DC compounds [26], resulting in minimal auxiliary effects. This fact is strongly supported by evidence provided by HRTEM images of Figure 9. Figure 9 accurately shows how the interface between the added TiB_2_ particles and α-Al is non-coherent, and no TiAl_3_ 2DC compound can be observed at the interface. This limitation contributes to the unsatisfactory refinement performance observed with external TiB_2_ particle addition.

As demonstrated earlier, in addition to the pure aluminum melt, these particles spontaneously form a coating layer at the leading edge of the growing grain boundary. Similar cases of particle-induced grain refinement have been reported in empirical studies [27,28]. This coating effectively impedes atomic diffusion and slows the growth rate during pure aluminum grain growth. Termed the physical growth restriction effect, this dual inhibitory mechanism is responsible for the observed reduction in grain size after TiB_2_ particle addition. However, the assembly of this coating with micrometer-sized particles may result in poor density, leading to the presence of irregularly shaped grains. Notably, irregular grains are not reported in grain refinement induced by nanoparticles [27,28,29].

A physical model of particle-induced grain refinement, focusing on spherical grains, has been developed [11]. In this experiment, the solidification conditions can be considered to approximate rapid solidification due to the instantaneous solidification time achieved by employing a steel mold. During the solidification process of the isothermal melt, the radius R of a diffusion-controlled growing spherical crystal can be obtained as follows [11,30]:

(3) $$R=\lambda \sqrt{Dt}$$

where D is the solute diffusion coefficient in the liquid, t is time, and λ is an interfacial parameter, determined by the invariant size approximation method:

(4) $$\lambda =[\frac{-k}{2\pi^{1/2}}]+{\left[\frac{k^{2}}{4\pi }-k\right]}^{1/2}$$

(5) $$k=\frac{2(C_{IL}-C_{0})}{C_{IS}-C_{IL}}$$

where $C_{0}$ is the solute content in the alloy melt and $C_{IL}$, $C_{IS}$ are the equilibrium interface compositions in the liquid and solid, respectively.

**Figure 10:**
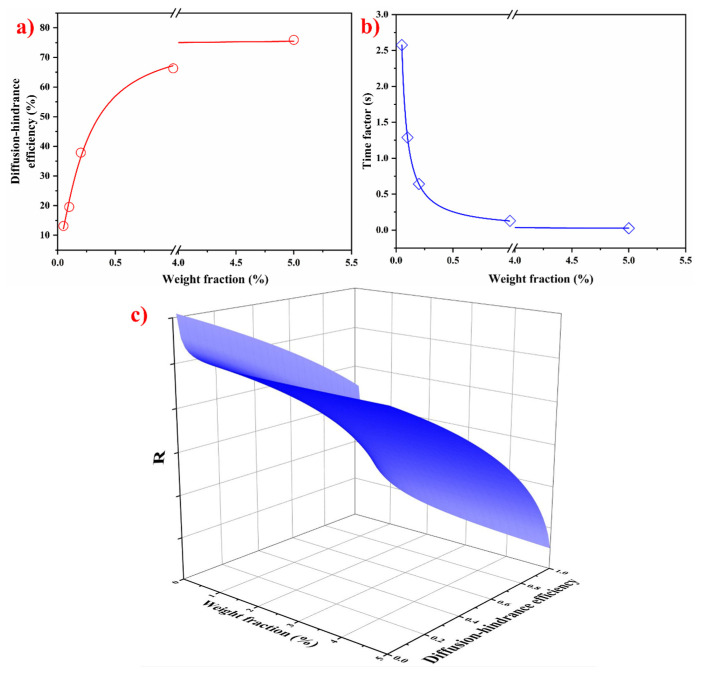
(**a**) Diffusion-hindrance efficiency versus particle addition level, (**b**) time factor versus particle addition level, and (**c**) illustration of R versus particle addition level and diffusion-hindrance efficiency.

Here, f is defined as the diffusion-hindrance efficiency resulting from the particle coating and τ is the time factor accounting for the characteristic time for particles to cover the growing crystal. The values of f and τ with respect to the weight fraction are plotted in Figure 10a,b according to their definition and expression based on the particle-induced adsorption model [31,32]:

(6) $$\tau =\frac{1}{C_{NP}K_{NP}}=\frac{d}{C_{NP}V_{NP}}$$

where d is the thickness of the particle layer, $C_{NP}$ is the concentration of particles in the aluminum melt, $V_{NP}$ is the kinetic velocity of particles in Brownian motion, and $K_{NP}$ is a constant.

(7) $$V_{NP}={\left(\frac{6KT}{\rho \pi d^{3}}\right)}^{\frac{1}{2}}$$

Here, K is the Boltzmann constant, T is the melt temperature, and ρ is the density of particles.

When t≫τ,

(8) $$R=\lambda \sqrt{\frac{D}{K_{NP}}C_{NP}^{-1}+(1-f)Dt}$$

The above analysis indicates that the diffusion-hindrance efficiency increases with the increase in the weight fraction of TiB_2_ added, while the time factor is the opposite. The current model provides a theoretical framework for understanding the impact of particles on grain refinement through the growth restriction imposed by the coating formed on the grain surface. As depicted in Figure 10a, there exists a direct positive correlation between diffusion-hindrance efficiency and grain refinement efficiency. This straightforward relationship enhances the practical applicability of particle-induced grain refinement, suggesting that increasing the addition level of TiB_2_ particles theoretically leads to a significant decrease in the grain size of pure aluminum. Additionally, when considering the time factor illustrated in Figure 10b, it becomes evident that the time factor decreases with the increasing particle addition level. Using a large number of additives can effectively shorten the time required to form coatings that can restrict the growth of α-Al grains. In other words, higher levels of particle addition expedite the process of particle self-assembly into a coating that restricts grain growth, thereby dynamically achieving effective grain size reduction. Figure 10c further illustrates the relationship between both the particle addition level and diffusion-hindrance efficiency. Remarkably, this figure confirms that grain size decreases with increasing levels of particle addition and diffusion-hindrance efficiency, which is consistent with the experimental observations (Figure 2 and Figure 3).

**Figure 11:**
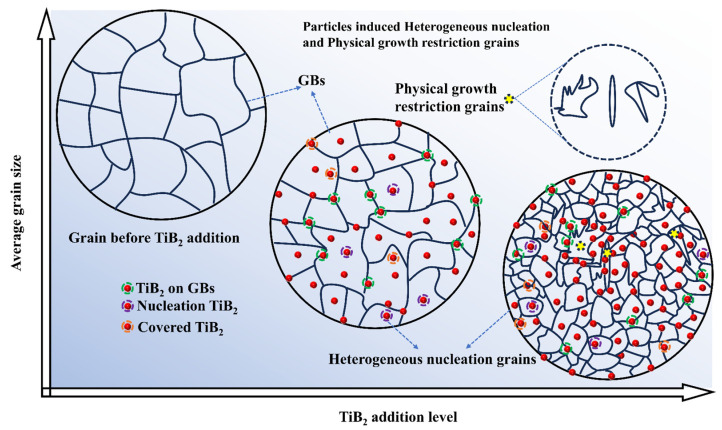
Schematic illustration of dual grain refinement mechanisms of pure aluminum induced by particles.

Figure 11 presents a schematic illustration of the dual grain refinement mechanism induced by particles, incorporating both heterogeneous nucleation and the physical growth restriction effect. Prior to the addition of TiB_2_ particles, the original grains of pure aluminum exhibited a coarse morphology typical of natural solidification. However, following the addition of TiB_2_ particles at a low level, as depicted in the central image, these coarse grains began to shrink in size through heterogeneous nucleation, with some TiB_2_ particles serving as heterogeneous nucleation sites within the grain’s interiors. While a certain number of TiB_2_ particles remained at the grain boundaries, their population was insufficient to exert the physical growth restriction effect. It is worth noting that although multiple TiB_2_ particles may be present within a single grain, only one typically serves as a heterogeneous nucleation site, while the remainder is covered and unavailable for further grain refinement. At higher addition levels, the substantial population of TiB_2_ particles exhibits self-assembly capabilities, forming a more complete coating driven by the solidification interface. At this stage, the combined effects of heterogeneous nucleation and physical growth restriction significantly contribute to grain refinement.

## 5. Conclusions

This paper presents the impact of externally added TiB_2_ particles on grain refinement in pure aluminum, with particular emphasis on the observed physical growth restriction effect following their addition. This introduces a novel approach for utilizing particles that do not participate in heterogeneous nucleation within the grain refinement process with master alloys, thereby opening avenues for the development of master alloys with high-grain refinement efficiency and practical applications. The summarized research findings are as follows:

1. External particles predominantly reside within the grains and at grain boundaries within the microstructure. Internal particles initiate heterogeneous nucleation, while particles located at grain boundaries form coatings.
2. Grain refinement induced by external particles primarily involves heterogeneous nucleation and physical growth restriction effects. This may result in irregularly shaped grains interlocking with each other alongside conventional polyhedral grains.
3. Regarding the physical growth restriction effect, increasing the amount of particle addition enhances the restriction’s effectiveness, leading to improved grain refinement efficiency and reduced time required for the formation of particle coatings.

## Data Availability

All available data supporting this publication are covered in this article.
